# Patient experiences of an electronic PRO tailored feedback system for symptom management following upper gastrointestinal cancer surgery

**DOI:** 10.1007/s11136-020-02539-w

**Published:** 2020-06-13

**Authors:** H. S. Richards, A. Portal, K. Absolom, J. M. Blazeby, G. Velikova, K. N. L. Avery

**Affiliations:** 1 Bristol Centre for Surgical Research, Bristol Medical School: Population Health Sciences, University of Bristol, Canynge Hall, 39 Whatley Road, Bristol, BS8 2PS UK; 2 Medical Research Council ConDuCT-II Hub for Trials Methodology Research, Bristol Medical School: Population Health Sciences, University of Bristol, Canynge Hall, 39 Whatley Road, Bristol, BS8 2PS UK; 3 Leeds Institute of Medical Research at St James, St James’s Hospital, University of Leeds, Bexley Wing, Beckett Street, Leeds, LS9 7TF UK

**Keywords:** Patient-reported outcomes, Qualitative, Surgery, Thematic analysis, Cancer, Electronic patient-reported outcomes

## Abstract

**Purpose:**

Complications following upper gastrointestinal (UGI) surgery are common. Symptom-monitoring following discharge is not standardized. An electronic patient-reported outcome (ePRO) system providing feedback to patients and clinicians could support patients and improve outcomes. Little is known about patients’ experiences of using such systems. This qualitative sub-study explored patients’ perspectives of the benefits of using a novel ePRO system, developed as part of the mixed methods eRAPID pilot study, to support recovery following discharge after UGI surgery.

**Methods:**

Patients completed the online ePRO symptom-report system post-discharge. Weekly interviews explored patients’ experiences of using ePRO, the acceptability of feedback generated and its value for supporting their recovery. Interviews were audio-recorded and targeted transcriptions were thematically analysed.

**Results:**

Thirty-five interviews with 16 participants (11 men, mean age 63 years) were analysed. Two main themes were identified: (1) reassurance and (2) empowerment. Feelings of isolation were common; many patients felt uninformed regarding their expectations of recovery and whether their symptoms warranted clinical investigation. Participants were reassured by tailored feedback advising them to contact their care team, alleviating their anxiety. Patients reported feeling empowered by the ePRO system and in control of their symptoms and recovery.

**Conclusion:**

Patients recovering at home following major cancer surgery regarded electronic symptom-monitoring and feedback as acceptable and beneficial. Patients perceived that the system enhanced information provision and provided a direct link to their care team. Patients felt that the system provided reassurance at a time of uncertainty and isolation, enabling them to feel in control of their symptoms and recovery.

**Electronic supplementary material:**

The online version of this article (10.1007/s11136-020-02539-w) contains supplementary material, which is available to authorised users.

## Introduction

Recovery from upper gastrointestinal (UGI) cancer surgery is associated with distressing and difficult symptoms that can occur for up to six months after hospital discharge [1–3]. Up to 50% of patients experience complications within one month of surgery [3–5], frequently after they have left hospital [6, 7]. Symptoms and complications during the initial recovery period can have negative impacts on health-related quality of life (HRQL) for up to 5 years post-surgery [5] and can range from pain, fatigue and nausea [8] to respiratory failure, sepsis and wound infections [9–12].

Patients are increasingly being discharged from hospital earlier following surgery, with an increased emphasis on recovery at home [13, 14]. Although earlier discharge can be feasible and safe [15, 16], patients’ symptoms are not usually routinely monitored post-discharge [17]. Evidence to support early discharge tends to focus on clinical outcomes rather than patient experiences [18]. Research suggests that patients find it difficult to obtain symptom management advice once they are at home [18]. Relevant verbal or written information is usually provided to patients pre-operatively or prior to discharge [19]. However, the shift towards earlier discharge may mean that current methods of pre-discharge education are no longer sufficient for effective patient self-management of symptoms [13]. Furthermore, incomplete understanding of patterns of recovery after discharge can cause patients to experience uncertainty and concern about how to recognise and respond to symptoms [13]. Uncertainty and a feeling of “going it alone” during recovery can have a significant detrimental impact on patients’ experience of recovery. Recent research shows, for example, that patients often report feelings of confusion and abandonment following discharge [20].

Electronic platforms to collect patient-reported outcome measures offer an efficient means for patients to report symptom data once they have left hospital. Monitoring of symptoms through the routine collection of electronic patient-reported outcome measures has been shown to enhance the detection of complications in cancer patients during treatment [21, 22]. Emerging evidence indicates that personalised electronic patient-reported outcome interventions providing tailored information post-discharge may lead to a quicker return to normal activities after surgery [23], improved HRQL and survival in cancer patients [24, 25]. However, there has been little qualitative research focusing on patients’ experiences of using such systems to support symptom management.

As part of the eRAPID project we have developed a novel electronic patient-reported outcome (ePRO) symptom-report and feedback system to improve recovery in patients who have been discharged from hospital following UGI cancer-related surgery. The development of the eRAPID system for use by breast cancer chemotherapy and pelvic radiotherapy patients has been described previously [26–28]. A prospective mixed methods pilot study has established the feasibility of the UGI surgery specific ePRO system, demonstrating that it is acceptable to patients and clinicians [26, 29]. Here we report the findings from the qualitative work that formed part of this pilot study, the aim of which was to understand participants’ experiences and perceptions of using the ePRO system after hospital discharge following UGI cancer-related surgery to support their symptom management.

## Methods

### The ePRO system

The IT elements include a patient-facing website incorporating a symptom-report questionnaire and enables secure transfer of data to EHR, allowing clinicians to view symptom reports [27]. All questionnaire items were selected from relevant EORTC modules. The development of symptom severity threshold algorithms and generation of feedback is described in detail elsewhere [26]. Briefly, scoring thresholds were developed and iteratively refined with input from clinicians (Cancer Nurse Specialists, Dietitians and Surgeons) and patients, and with data from qualitative interviews with Cancer Nurse Specialist and quantitative data from completed patient self-report questionnaires. The ePRO system provides tailored feedback to patients based on the severity of their reported symptoms to the online symptom-report questionnaire. Feedback is dependent on symptom severity and includes symptom self-management advice appropriate to their stage of recovery or advice to contact health care professionals (HCP) if symptoms are clinically concerning. The system is integrated into hospital electronic records, enabling clinicians to access real-time individual symptom reports and graphs. When concerning symptoms are reported, clinicians receive an automated alert email instructing them to review symptom reports and contact the participant. Participants are provided with graphs illustrating how their individual symptoms change over time. The types of feedback generated by the ePRO system are illustrated in Table 1.

**Table 1:** Guided patient management by symptom severity within the ePRO system [26]

Symptom severity level	ePRO system action/advice	Example of ePRO action or advice for shortness of breath
Level 0: minimal/no symptoms	No patient advice required	Thank you for completing the questionnaire
Level 1: expected symptom(s)	Patient advice: self-management advice^a^	Some shortness of breath after physical activity such as climbing the stairs is a normal part of recovery. You may wish to consider the advice below…
Level 2: potentially concerning symptom(s)	Patient advice: contact a healthcare professional today if symptom is new or has not already been reported	If you have not already discussed your shortness of breath with your medical team we recommend that you contact your CNS team today to discuss your symptoms
Level 3: symptom(s) indicative of a complication	(i) Patient advice: contact a healthcare professional immediately (ii) Clinician alert: automated email to a Cancer Nurse Specialist	We recommend that you contact the hospital now to discuss your symptoms with the medical team. If you are unable to contact the CNS team, please call your GP to discuss your symptoms today

^a^Patient self-management advice was developed from NHS patient information leaflets and in close conjunction with patients and clinicians.

### Participants

This mixed methods prospective pilot study was conducted at Bristol Royal Infirmary University Hospitals Bristol NHS Foundation Trust. Consecutive patients who had undergone UGI surgery between August 2017 and March 2018 were screened for eligibility from inpatient clinic lists by a hospital research nurse. Eligibility criteria included patients who had undergone UGI cancer-related surgery, had access to a computer/mobile device and the internet at home, were ready for hospital discharge to their home, were over 18 and were fluent in English. Participants were eligible for inclusion if they had undergone oesophageal gastric (e.g. oesophagectomy, gastrectomy) or hepato-pancreato-biliary (e.g. Whipples, hepatectomy) surgeries. These criteria were established based on developmental work that identified similar patient recovery and clinical care pathways for these patient groups [26].

### Data collection

The data for this qualitative study were collected as part of a wider mixed methods pilot study. Patients were approached by a research nurse when they were ready for discharge. Eligible patients were given a participant information leaflet and the opportunity to ask questions, and those wishing to participate provided written informed consent. Participants were asked to complete the ePRO system questionnaire twice in the first week and weekly for eight weeks post-discharge and all were asked to take part in telephone interviews at these timepoints. All interviews were conducted by HR and/or AP. HR is an experienced mixed methods Senior Research Associate and AP is a Research Nurse and Cancer Nurse Specialist. Quantitative data relating to ePRO system response rates, symptom data and clinical outcomes were collected and are reported elsewhere [29].

### Weekly telephone interviews

Semi-structured telephone interviews were conducted weekly for eight weeks to coincide with completions of the ePRO system. All participants were interviewed regarding their use of the ePRO system and, in accordance with the principles of targeted transcription [32], only those interviews in which participants discussed using the ePRO system within the context of symptom management were transcribed for qualitative analysis. For example, data from weekly interviews where participants had not experienced symptoms or where only yes/no responses were obtained were not transcribed (see “Data analysis” section). Telephone interviews generally took place when the participant was at home and all participants consented to audio recording.

Interviews focussed on participants’ experiences of using the ePRO system and the type of feedback they had received. The interview guides were adapted from pilot work relating to the development of the ePRO system [26] and is provided in Online Appendix 1. Participants were asked about how suitable they found the advice they had received. For example, when participants had received a prompt to contact an HCP, they were asked whether (and reasons why) they had or had not made contact and what the outcome of any contact had been. AP and/or HR made notes during telephone interviews, and this data was used to contextualise the interview transcripts during coding. Weekly interviews were generally 5–20 min in duration.

### End-of-study interviews

A subset of approximately 10% of participants were invited to a face-to-face interview once they had completed the 8-week follow-up period. Participants were selected in order to represent a range of patient experiences of using the system, including those who had fully or partially engaged with the system, and those who had and had not experienced post-discharge complications. These interviews were conducted and audio-recorded in the participant’s homes by HR and AP. End-of-study interviews were of 1–1.5 h in duration.

### Data analysis

Thematic analysis was conducted in accordance with Braun & Clarke’s guidelines [30]. Thematic analysis is a flexible and widely used method for analysing qualitative data, by which underlying themes and concepts are identified, derived and interpreted within the wider context of the data. Thematic analysis was chosen because the research question focused on patients’ perceptions and experiences of using the ePRO system within the context of their symptom management at home, as well as within established clinical care pathways. Thematic analysis provides a theoretically flexible approach useful for such pragmatic situations where it is necessary to contextualise findings, within both the experiential and interpretative realities of the participant, and the more positivist scope of an existing healthcare system [31]. All end-of-study interviews were transcribed verbatim. Audio recordings of all weekly interviews were reviewed several times by HR and AP. While it was not feasible to transcribe all of these interview transcripts verbatim, only those weekly interviews in which participants discussed data of relevance to the research question (i.e. received symptom management advice from the ePRO system) were transcribed verbatim. This form of targeted transcription is recognised as a pragmatic means of analysing large numbers of interviews by focusing on data most relevant to the research question [32]. HR and AP discussed and identified interviews relevant for verbatim transcription. HR was familiarised with the data by re-reading transcripts multiple times. Descriptive codes were identified within the data and organised using NVivo 12 software. Codes were examined and interpreted to identify overarching themes which were reviewed within the context of the data using methods of constant comparison. HR coded all the data and themes were reviewed by KA for coherency and consistency with the data. Regular meetings were held with the core study team (HR, AP & KA) to discuss emerging findings. Analysis was conducted until thematic saturation was reached and no new themes were emergent from codes [33]. As this was a pragmatic feasibility study and the qualitative data were collected as part of a wider mixed methods study it was not feasible to invite participants to comment on findings.

## Results

In total 109 patients were screened for eligibility, of which 41 (38%) were eligible and invited to participate, and 29 (71%) consented (mean age 64 years, standard deviation (SD) 9 years, range 43–81, 19 men). All 29 participants were interviewed at least once following discharge. Seven participants withdrew from the study because they felt too tired or unwell to continue (*n* = 6) or due to a prolonged hospital readmission (*n* = 1). An additional six participants took part in telephone interviews but did not provide data relevant for analysis. As such these interviews were not included in the analysis. The analysis reported here focuses on data collected from 35 weekly and three end-of-study interviews carried out with the remaining 16 study participants. Fifteen participants received ePRO advice to contact an HCP relating to concerning symptoms and three were readmitted to hospital following discharge due to adverse events. The frequency of reported symptoms, ePRO system actions, hospital readmissions and clinical outcome data for the full pilot study are reported elsewhere [29]. Participant demographics and clinical details are reported in Table 2.

**Table 2:** Participant demographics

	Participants (*n* = 16)
Sex, *n* (%)	
Male	11 (69)
Age, years	
Mean (SD)^a^	63 (10)
Range	43–73
Ethnicity, *n* (%)	
White British	11 (69)
Chinese	1 (6)
Not stated	4 (25)
Cancer diagnosis, *n* (%)	
Yes	9 (56)
Length of hospital stay, days	
Mean (SD)^a^	13 (10)
Range	3–35
Surgical procedure received, n (%)	
Oesophago-gastric resection	6 (38)
Hepatobiliary resection (inc. Whipples)	10 (62)
Marital status, n (%)	
Married/civil partnership/cohabiting	14 (88)
Single	1 (6)
Widowed	1 (6)
Education, *n* (%)	
Further education	14 (88)
Degree/professional qualification	10 (62)
Employment status, *n* (%)	
Retired	8 (50)
Working full-time	5 (31)
Working part-time	2 (13)
Not in paid employment	1 (6)
Computer usage, *n* (%)	
Daily	15 (94)
Weekly	1 (6)
Proficiency with computer, *n* (%)	
Easy	14 (88)
Sometimes difficult	2 (12)

Participants described the ePRO system to be quick, easy and straightforward to use, confirming the findings from the pilot study [26]. All participants described the ePRO system as a positive adjunct to their recovery. Although the ePRO measure was developed from a widely used and validated questionnaire [26], some participants discussed factors commonly associated with questionnaires, such as the inflexibility of response options, the repetitive nature of answering questions repeatedly and receiving the same outcome/advice multiple times. Despite these observations, participants felt that these were unavoidable issues for all questionnaires that did not adversely impact on their experiences of using the system.

**Fig. 1:**
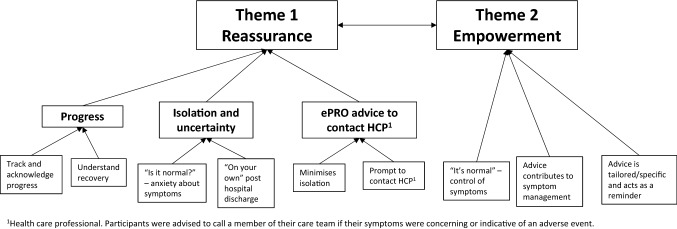
Emergent themes from thematic analysis of patient interviews

Two overarching themes were identified from the thematic analysis: *Reassurance* and *Empowerment*. Figure 1 provides an overview of interrelated and independent themes and sub-themes. Table 3 provides examples of patient quotations supporting the identified themes.

**Table 3:** Overview of themes from thematic analysis of patient interviews

Themes, sub-themes and examples of codes	Example quotes
Theme 1: Reassurance ePRO advice to contact HCP Prompted contact with HCPs; minimises isolation; enables appropriate choices in contacting HCPs; reassurance gained from HCP contact; clinical input following HCP contact	PT 1882 “I wouldn’t know whether to contact anybody or not” PT 1213 “I’d already called on Tuesday…I suppose I would have [called an HCP] if I hadn’t have done it already” PT 1230 “I did speak to the GP just about a little bit of wound drainage, and quite reassuring really” PT 1882 “So I got in touch with my GP and she said she’s going to put me on different antibiotics”
*Isolation and uncertainty* Isolation post-discharge; ePRO provides link with HCP; uncertainty about symptoms and expectations of recovery; lack of recall of guidance/advice given pre-discharge; anxiety about ‘normality’ or symptoms	PT 1230 “I worry, I don’t want to bother anybody, so until [advice from ePRO to contact HCP], yeah it’s weird” PT 1242 “Sometimes you feel you shouldn’t ring your nurse, you know” PT 1213 “I think anybody in my position would be a bit worried about the diarrhoea, you know if somebody can reassure you that this can happen…and then it makes you feel better, [otherwise] you just think ‘Oh what am I doing wrong?’…If you read that [ePRO advice] first…that it’s normal for a bit, but if it lasts you’ve got to get in touch with somebody” PT 1226 “I suppose in a sense it reminds me not to worry over much about feeling a bit tired…because you know it’s there and it’s a natural consequence of what’s happened…because it’s ‘don’t worry about it, this is what happens, it’s normal.’ So in that way I found it reassuring”
*Progress* Impact of graphs; enables participants to track and acknowledge improvements in symptoms; accurate reflection of symptom experiences; improves understanding of the process of recovery	PT 1237 “It helps you to see that obviously they’re expecting you to possibly have this or possibly have that [symptom]. And also…looking at the graph[s] and looking at my previous answers and seeing how they’ve changed, and interestingly as I said those euphoric first answers where everything was absolutely wonderful [when I was] still on tramadol and goodness knows what else. And then the reality of how I really am and then slowly seeing the recovery over that time has been very helpful” PT 1242 “It’s probably the first time I’ve looked at it and thought that’s good, because I had a few wobbles the last couple of weeks, I had an infection, so yeah it was quite encouraging, cuz [sic] I do feel quite a lot better” PT 1226 “I find it reassuring when I look at the graphs having completed it all…I find that it actually gives a pattern which reinforces how I feel about what has happened since the op”
Theme 2: Empowerment Advice is tailored and specific; advice is a reminder of what to do and of information previously given by HCPs; directly contributes to symptom management; eases anxiety by confirming symptoms are ‘normal’; enables participants to feel more in control of their recovery	PT 1208 “I think with the pills and with the advice on the [ePRO system] I feel in control of things” PT 1226 “It just showed me that it was exactly the right thing to do…Because I think it helps towards recovery, rather than just dealing with it myself” PT 1213 “[ePRO advice] is talking about what you can do, what’s good to help you. Whereas the hospital website is more or less telling you this is what happens” PT 1224 “You can then look at the suggested actions you can take to alleviate those symptoms” PT 1208 “It’s all the guidance which the doctors told me and what [ePRO] tells me as well. So it’s a constant reminder to obey the rules”

## Theme 1: Reassurance

Participants described the ePRO system as reassuring for several reasons relating to uncertainties about the clinical significance of symptoms, isolation experienced following hospital discharge, and providing a means of tracking the progress of their recovery. These sub-themes are detailed below.

### Isolation and uncertainty after discharge

By providing advice to contact their care teams, the ePRO system acted as a link to HCPs. Some participants felt that they were “on their own” once they were at home. This was confounded by patients’ uncertainties regarding the causes, meaning and significance of the symptoms they were experiencing, and at what point they should contact HCPs. In this context, ePRO reassured them that contacting HCPs was an appropriate decision.

PT 1224 “So [ePRO system] makes you feel not cut off…Because otherwise I think the issue can be you get all this attention in hospital, and then suddenly that’s it – you’re off on your own.”

Some participants felt uncertain about what symptoms they were likely to experience and what to expect during their recovery. Many attributed these feelings to a lack of information provision prior to discharge.

PT 1209 “When I first came home, I was a bit surprised by the fact that I wasn’t really given much in the way of guidance. As to what to expect, in terms of…the symptoms I might experience when I came home, and what I should or shouldn’t do…”

For some, this led to anxiety regarding whether their symptoms were ‘normal’ for their stage of recovery, leading them to question how well they were managing. In these instances, the ePRO self-management advice reassured participants that their symptoms were typical.

PT 1208 “[The ePRO system] confirms that in the [tailored feedback] that that’s normal for this period of time in your development so I find that, the [tailored symptom advice] at the end quite reassuring.”

### ePRO system advice to contact HCP

Participants reported barriers to contacting HCPs, including uncertainties regarding whether their symptoms were clinically concerning. Many described the ePRO system prompts as reassuring by supporting them to overcome these barriers. Some participants were confident about recognising severe symptoms (e.g. fevers and infections) but were unsure if they should contact HCPs regarding more ambiguous symptoms such as pain, fatigue and wound problems. Although participants were aware of how to contact HCPs, ePRO system prompts gave them confidence that their symptoms warranted contacting their care team.

PT 1219 “I spoke to the [cancer nurse specialist] and she put me in touch with the dietitian. But that all followed on from my completing a questionnaire, so it prompted me to do it I think.”

While ePRO system feedback acted as a prompt to contact HCPs, data from weekly interviews with Cancer Nurse Specialists (reported elsewhere, Richards [29]) suggests that it did not result in participants making additional unnecessary calls to their care teams. When participants were already in contact with HCPs and felt that their symptoms were being appropriately managed, or had upcoming clinical appointments, many reported that they did not make additional calls to clinicians.

PT 1224 “Well as I had an appointment…this morning…I didn’t see any need to call anybody.”

When participants did contact HCPs following ePRO system feedback, this often resulted in clinical interventions such as additional appointments or new prescriptions, or additional reassurance and advice.

PT 1213 “[The cancer nurse specialist] said I’m going to ring your GP…and they rang me back and said we’ll do a blood test.”

### Progress

For many participants, using the ePRO system enabled them to see recovery as a process instead of focussing on the individual experiences of distressing symptoms. The system provided relevant and useful advice for managing their symptoms, and a means of monitoring their progress during recovery. Participants reported that the individual symptom graphs generated by the ePRO system accurately reflected their experiences and described these as beneficial for tracking recovery. Graphs confirmed that they were improving and provided a means of acknowledging how their symptoms had changed since hospital discharge, enabling them to observe patterns and understand their symptoms in context.

PT 1226 “You don’t see them in isolation as one week, you see [graphs of symptom reports] as a pattern of progress and that’s the way to look at them…when I look along the row from where I was to begin with to now where most [symptoms] are at zero I’m pleased.”

## Theme 2: Empowerment

The ePRO system enabled participants to appropriately manage their symptoms and feel more in control of their recovery and health. In this way, reassurance provided by the ePRO system empowered participants’ in their recovery, providing advice relevant to their symptoms that reflected their experiences. Participants talked about following the advice (e.g. food portion size, pain management and activity pacing), and how this helped them better manage their symptoms and recovery.

PT 1224 “[When] you’re having a bit of difficulty you can then look at the suggested actions [and tailored advice] you can take to alleviate those symptoms.”

The advice confirmed whether participants’ symptoms were typical, eased their anxiety and increased their confidence in their ability to manage their recovery.

PT 1226 “I go through the questionnaire and by the time I’ve finished…I’m comfortable in my own mind, and therefore I don’t need to go searching elsewhere [online for information about symptoms]…I actually don’t worry unnecessarily…[the tailored advice] just showed me that it was exactly the right thing to do…Because I think it helps towards recovery, rather than just dealing with it myself.”

The usefulness of the ePRO system advice was underpinned by participants’ feelings that it was tailored specifically for their symptoms. Participants felt like it was “aimed” at them, and that the advice was appropriate and achievable.

PT 1208 “At the end it all sort of comes together and it gives you advice…It works well…and aimed at me specifically.”

The ePRO system enhanced information provision by ensuring participants had instant access to relevant symptom self-management advice. For many participants, the ePRO system advice prompted them to recall information they had received from clinicians earlier on in their recovery, but which they had subsequently forgotten. This was the case for symptom management strategies, and for prescribed medications.

PT 1242 “I must have read it [patient information leaflet] in the first week or so and I’d forgotten all about it. Yeah…[I’ll] try to follow that [ePRO system] advice…It’s reminded me and [I’ve] thought I have got some medication for that, I should be taking it.”

## Discussion

This study explored patients’ experiences and perceptions of using an electronic symptom-report and feedback system to improve recovery following UGI cancer-related surgery. The ePRO system provides tailored self-management feedback depending on the severity of reported symptoms. Participants reported that the ePRO system enhanced their recovery at home by providing reassuring advice regarding their symptoms and when to contact HCPs, thereby empowering patients to manage their recovery.

A key theme was the reassuring role of the advice to contact HCPs in participants’ decision-making. Some participants were unsure about whether they should contact HCPs, often because of uncertainties relating to the relative severity of their symptoms and an unwillingness to “bother” clinicians. Uncertainties about contacting HCPs outside of routine appointments has been associated with delays in help seeking [34] which can lead to delays in the detection of AEs following surgery [35]. Although participants in this study knew how to contact their care team, being prompted by the ePRO system encouraged them to do so. Other studies have demonstrated that uncertainties about what to expect post-discharge can result in patients and carers expressing a constant need for reassurance from HCPs [13]. However, by providing relevant and reassuring guidance to participants about when it was appropriate to contact HCPs, or when symptoms could instead be self-managed, the ePRO system enabled participants to make informed decisions. Similar themes of reassurance and increasing confidence in decision-making have been reported by participants using the eRAPID system during chemotherapy treatment [36].

Consistent with previous research, participants felt that they lacked information about symptoms management necessary to support them during their recovery [18, 37], and experienced uncertainties and isolation following discharge from hospital [13, 20]. Doubts regarding how to obtain advice during recovery can result in feelings of vulnerability and anxiety [18]. In this study, feelings of isolation were buffered to some extent by engagement with the ePRO system, with participants describing it as a link to HCPs and to relevant symptom management advice. The tailored advice enabled participants to gain more understanding of what to expect from their symptoms and during recovery. Accurate expectations of recovery after major cancer surgery have been shown to have a positive impact on patients, and can reduce worry and negative thoughts [38]. Surgery patients have reported anxieties regarding what they perceived to be insufficient information provision and preparation prior to hospital discharge [13]. Indeed, it is well documented that patients are often unable to recall medical information provided to them by clinicians [39–43]. This effect is confounded in surgical patients by the cognitive impairments associated with anaesthesia, intensive care treatment [44] and symptoms such as fatigue, pain and sleep deprivation [45], all of which can negatively affect information retention and accurate recall. Electronic methods of delivering personalised, real-time self-management education for surgical patients can overcome some of these barriers [45], improve shared decision-making [46] and improve post-operative health outcomes [47].

Patient empowerment can be described as “the patients’ subjective sense of control over their own disease and treatment management” [48]. Participants in this study described the ePRO system advice as helping them to maintain a sense of control over their recovery. This reassurance enabled them to acknowledge improvements in their symptoms over time and reduced their anxiety about identifying ‘normal’ symptoms for their stage of recovery. Similarly, improvements in HRQL have been associated with patients’ perceived ability to take control of their recovery instead of feeling that their symptoms are controlling their lives [38]. Participants’ reported that, by providing tailored and reassuring advice about self-management strategies and guidance on when to contact HCPs, the ePRO system empowered them, helping them to address feelings of isolation and uncertainty often experienced by surgical patients after leaving hospital.[18, 20, 38].

This study has several limitations that should be considered when interpreting the results. This was a single centre study of a specific patient group, which may potentially limit the transferability of these findings to other groups. In this cohort, incidence of post-operative severe AEs was relatively low. This may indicate that these participants had more favourable experiences of recovery. Although interview data from three participants who had been readmitted to hospital due to adverse events were included in this analysis, it was not possible to obtain qualitative data from all participants who became very unwell or were readmitted due to e.g. participant withdrawal or prolonged readmissions. Future work should focus on this group of patients to determine the extent to which such electronic symptom reporting systems are feasible for patients who develop severe complications. Targeted transcription is a cost and time-effective approach to analysing qualitative data; however, it does introduce a risk that not all relevant data were analysed. To reduce this risk, several team members reviewed audio files to ensure all pertinent data was transcribed. In addition, it is important to note that only data from participants who received feedback from the ePRO system and those who completed the study were analysed. Understanding the views of those who withdraw or did not fully engage with the ePRO system could have provided some additional insights, which may have differed from those reported here. Additionally, all electronic systems pose a potential barrier for engagement amongst patients due to language, literacy and access to electronic devices. As eligibility criteria for this study included fluency in English and home internet access, findings are not transferable to groups outside of these criteria.

These findings have implications for clinicians and policy makers in terms of enhancing the provision of symptom management information for patients and empowering them to feel more in control of their recovery at home. The ePRO system was found to be feasible and useful for patients. A multicentre RCT is planned to examine to effectiveness of the ePRO system in UGI cancer-related surgery patients.

## Conclusion

Upper gastrointestinal cancer-related surgery patients found the ePRO symptom-monitoring and feedback system to be acceptable and reassuring during their recovery at home. Participants’ reported that, by providing self-management advice tailored to individual symptoms, the ePRO system addressed their anxieties and uncertainties relating to their recovery. Patients described feeling more in control of their recovery while using the ePRO system and experienced it as a link to their care teams. Participants’ felt that the ePRO system enhanced patient information provision relating to management of symptoms, by providing real-time access to advice and improving recall of guidance provided to them while in hospital.

## Electronic supplementary material

Below is the link to the electronic supplementary material.

Supplementary file1 (DOCX 18 kb)
