# Supplementary material for: Patient experiences of an electronic PRO tailored feedback system for symptom management following upper gastrointestinal cancer surgery
Source: Qual Life Res. Author manuscript; Available in PMC 2021 Nov 1. (PMC8528794; doi:10.1007/s11136-020-02539-w)
Supplement: Appendix 1 [file EMS128232-supplement-Appendix_1.docx]

**Appendix 1: Interview Topic Guides.**

**Weekly interviews**

Primary aim:

1. To understand participants’ experiences of using the eRPO system for symptom management during recovery at home following UGI cancer-related surgery.
2. Interviews were conducted weekly by telephone to coincide with completion of the ePRO.
3. **eRPO questionnaire usage**
   1. Have you completed the ePRO questionnaire in the last week?
   2. How was completing the ePRO questionnaire this week? Did you understand the questions?
   3. Did the ePRO system advise you to call your medical team about your problems? Did you call your medical team after eRAPID advised you to? If no, why did you choose not to call them? If yes, who did you speak to?
   4. What happened when (if) you phoned your medical team? Were you given any advice over the phone? What was that like?
   5. Did the online system give you any feedback or advice about your symptoms? If yes, what symptoms did you get advice about? How was this advice?
   6. Do you think the advice was relevant to your symptoms? Did you do anything differently after you read this advice?
   7. Were there any symptoms you WANTED advice about which the system didn’t show you advice for?
   8. Do you have any other comments about the eRAPID online system?
4. **Healthcare usage**
   1. Did you have any telephone contact with any health care services in the last week? If yes, who were you in contact with, for what reason and what was the outcome?
   2. Did you see any health care professional in person in the last week? If yes, who were you in contact with, for what reason and what was the outcome?

**End of study interviews**

Primary aim:

1. To gain an in-depth understanding of participant’s experiences of using the ePRO system.
2. Interviews were conducted with a sub-set of approx. 10% of participants following completion of the eight-week follow up period.
3. Can you tell us what it was like when you first came home from hospital? Did you have any readmissions/clinical problems?
4. What was being in the study like?
5. Did you get any feedback from the questionnaire?
6. Did it advise you to call anyone about your symptoms? What happened?
7. Did it give you any advice about how to manage your symptoms? What was that like?
8. Did the advice feel relevant/helpful/useful? Could it have been better?
9. Was there any advice or reassurance you wanted, or you think would have benefited you, which you didn’t get from the questionnaire?
10. What made you want to complete the online questionnaire?
11. Where there any things which made you NOT want to complete the online questionnaire?
12. We sent you reminders to complete the eRAPID questionnaire every week. What was that like?
13. Did you ever complete the questionnaire at extra times, i.e. more than once a week?
14. What did you think about filling it in weekly?
15. Did you ever have any help from other people to complete the online questionnaires?
16. Did you ever have any problems accessing the online questionnaire?
17. Are you glad that you took part in the study?
18. Do you think that being in the study made a difference to your recovery?
